# Case of Orbital Cysticercosis Presenting as Recurrent Cellulitis Diagnosed on Multi-Modality Imaging

**DOI:** 10.7759/cureus.18242

**Published:** 2021-09-24

**Authors:** Nikhith Soman, Rachit Khandelwal, Sagar Maheshwari

**Affiliations:** 1 Radiology, Dr. D.Y. Patil Medical College, Research Centre and Hospital, Pune, IND

**Keywords:** recurrent orbital swelling, orbital cysticercosis, superior rectus muscle, b-scan ultrasonography, role of mri

## Abstract

Cysticercosis results in humans when infected with the larval stage of taenia solium which is called cysticercus cellulosae. The target organs usually involved are the brain, eyes, spine, and skeletal muscles. The ocular form of cysticercosis can affect the intra-ocular structures or involve the orbital adnexa. Intraocular involvement is relatively common and is readily diagnosed owing to its obvious visibility on a basic slit-lamp examination, however, affection of orbital adnexa is infrequent. Moreover, solitary involvement of one of the extraocular muscles is rare and difficult to diagnose as it presents with a spectrum of non-specific symptoms. We report a rare case of orbital cysticercosis with a solitary left superior rectus muscle involvement, who presented with recurrent on and off lid swelling extending for two years with double vision and restriction of downward gaze.

## Introduction

Cysticercus cellulosae, the larva of taenia solium is the causative organism for human cysticercosis. It can affect a wide range of systems with the target organs being the brain, eyes, spine, and skeletal muscles. Amongst these neurocysticercosis is relatively common when it manifests as multiple space-occupying lesions within the brain parenchyma at the grey-white matter interface. The ocular involvement occurs in two forms: intraocular and orbital adnexal [1-3]. The intraocular form includes the involvement of anterior or posterior segments of the eye while extra-ocular muscles, subconjunctival space, eyelid, optic nerve, and retro-orbital space are involved in the orbital adnexal form. The occurrence of ocular cysticercosis with no other systemic involvement is rare and isolated orbital adnexal involvement is an occasional occurrence in clinical practice [4].

In our medical practice, a patient presented with a history of recurrent on and off episodes of lid swelling for two years who was being managed conservatively associated with double vision and restricted eyeball movements in downward gaze. A diagnosis of isolated cysticercosis of superior rectus muscle was made on B-Scan ultrasonography and confirmed on MRI scan.

## Case presentation

A 25-year-old female presented with recurrent left eyelid swelling for two years which was being treated conservatively, however no definite resolution of symptoms was achieved. During the period of repeated episodes of cellulitis, the patient was suggested a routine follow-up of 6-8 weeks after presentation and emergent follow-up in an event of aggravation of symptoms. The patient was a non-vegetarian by diet and had occasional history of consumption of meat from food vendors. However, no accurate history of consumption of undercooked/baked meat was elicited. No history of any comorbidities like diabetes mellitus or neurological insults. On examination, there was associated diplopia with difficulty in downward gaze. There was no associated loss of vision. Since a suspicion of extra-ocular muscle involvement was raised on examination findings, other complications in the form of proptosis, ptosis, double vision, strabismus and eventual vision loss were taken into consideration.

Laboratory investigation showed mildly raised eosinophils, with the rest of the parameters within normal limits. Initially, an Ultrasound B scan of both eyes was performed and was followed by an MRI contrast of the brain with orbits.

**Figure 1:**
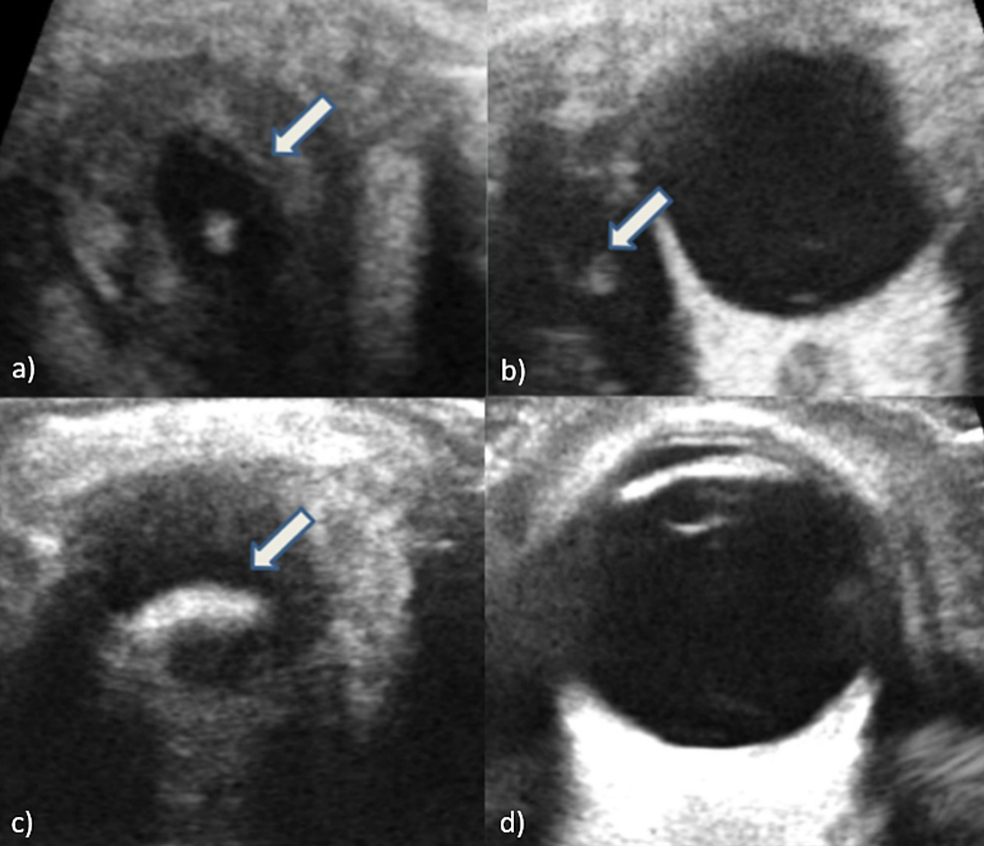
Ultrasound B scan showing a well-defined cystic lesion with an echogenic mural nodule (white arrows) in the superior rectus muscle of the left eye. (a) Transverse view just superior to the section of the eyeball showing a well-defined hypoechoic cystic lesion with a tiny echogenic mural nodule as its content within the superior rectus muscle. (b) Longitudinal view showing that the lesion is cranial to the eyeball separate from the intra-ocular compartment. (c) Transverse view of the superior rectus showing thickening and bulky muscle with peri-lesional fat stranding. (d) Transverse view of the intra-ocular compartment reveals a normal appearance with no focal lesion within.

Ultrasound B scan was done and it showed a well-defined cystic lesion with an echogenic mural nodule in the superior aspect of the left eye, most likely in the superior rectus muscle (Figure 1). Mild left proptosis was noted.

**Figure 2:**
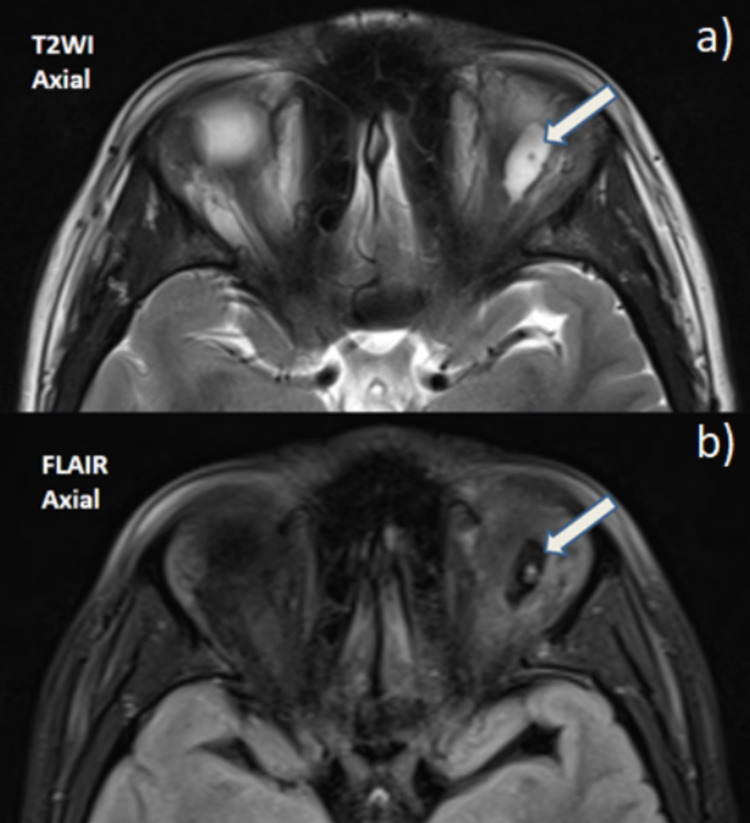
(a) On axial T2WI a focal hyperintense lesion is noted within the superior rectus muscle with a tiny eccentric hypointense mural nodule. (b) On axial FLAIR sequence, the hyperintensity observed on T2WI was suppressed.

Subsequently, MRI scan revealed a well-defined oval cystic lesion in the left superior rectus muscle measuring 15 x 8 x 6 mm. The lesion was hypointense on T1WI, hyperintense on T2WI, and suppressed on FLAIR images (Figure 2). No foci of blooming were noted on GRE images and no there was no evidence of diffusion restriction within the lesion. It showed subtle peripheral contrast enhancement.

**Figure 3:**
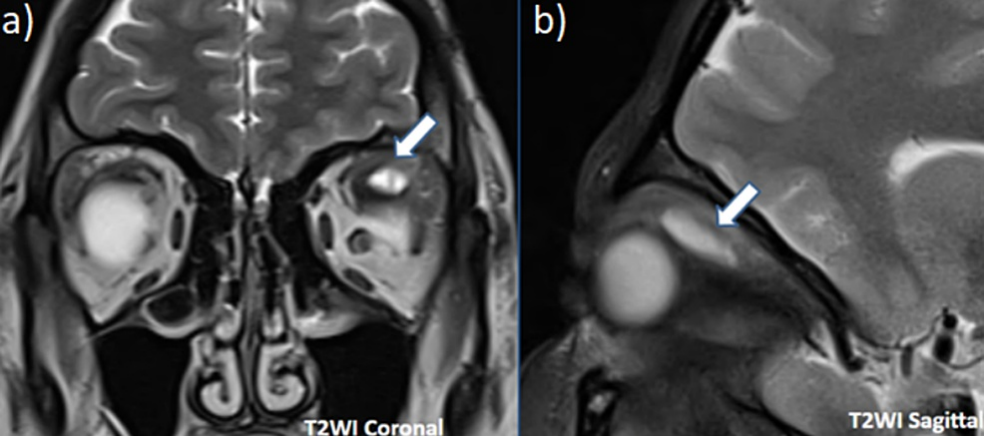
(a) Coronal T2WI reveals soft tissue inflammatory infiltrates around the hyperintense lesion with bulky appearance of superior rectus muscle in relation to other extra-ocular muscles. (b) Sagittal T2WI denotes the fusiform enlargement and thickening of the entire superior rectus muscle.

An eccentric focal T1 hyperintense mural nodule was noted within the cyst, appearing hypointense on T2WI and showing mild post-contrast enhancement. The left superior rectus muscle showed diffuse thickening and appeared bulky with surrounding soft tissue infiltrates (Figure 3). Intense post-contrast enhancement was noted in post-contrast images. 

**Figure 4:**
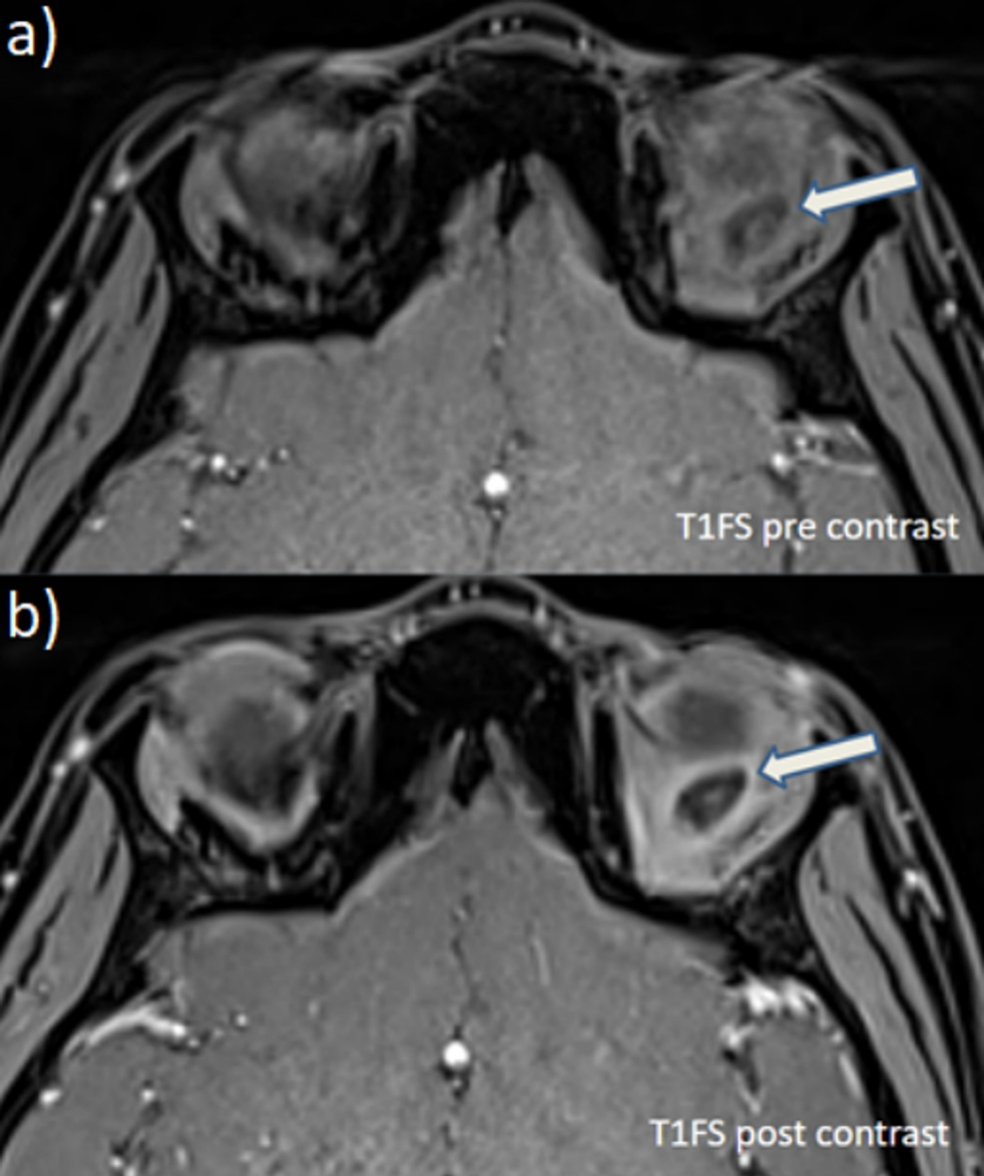
Comparing the T1FS pre-contrast images (a) with post-contrast images (b) we observe that the cyst showed subtle peripheral enhancement, while the nodule within the cyst showed mild enhancement. Surrounding soft tissue infiltrates show heterogeneous post-contrast enhancement.

The surrounding soft tissue infiltrates showed heterogeneous post-contrast enhancement while the cyst showed mild peripheral enhancement (Figure 4).T2/FLAIR hyperintensities were noted in the subcutaneous plane in the left periorbital region in the preseptal compartment and retrobulbar region - suggestive of cellulitis. The brain parenchyma revealed no obvious focal lesions.

These findings were conclusive of cysticercosis affecting the orbital adnexa with involvement of the superior rectus muscle with cyst likely in the vesicular stage. However, no evidence of concurrent neurocysticercosis was seen.

Following the diagnosis, the patient was started on oral albendazole (a broad-spectrum antihelminthic) at a rate of 15 mg/kg body weight for a period of four weeks following which she showed complete resolution of symptoms.

## Discussion

Cysticercosis is caused by the larval form of taenia solium, it is a systemic parasitic disease. It is commonly observed in regions with improper sanitation and is prevalent in countries of South East Asia, the Indian sub-continent, Mexico, South America, and sub-Saharan Africa [5]. Other countries where orbital form of cysticercosis has been reported are: Mexico, Latin America, China, Indonesia and Eastern Europe. In countries of Eastern Europe particularly Portugal, the cases are generally found to be as a result of travel and immigration [6].

Risk factors associated with spread of T.solium infection include sanitary factors, breeding of pigs, consumption of undercooked meat, a positive family history of parasitic infestation, travel history to an endemic areas and any visitor from endemic countries [7].

The larval form, i.e., cysticercus cellulosae has particularly three stages in the disease progression. The live/vesicular stage is characterized by a living cyst and a well-defined scolex. Usually minimal or no inflammation is observed in the surrounding tissues in this stage. In the colloid vesicular stage, the larva begins to die and the cyst wall is no longer intact which leads to inflammatory changes in surrounding tissues due to released toxins. Following larval death, it is resorbed or may be calcified in the final stage called a calcified nodular stage [8].

Cysticercosis affects the eye and depending on the site of affection it is divided into two forms: intra-ocular (anterior or posterior segment of eye) and orbital adnexal (extra-ocular muscles, subconjunctival space, eyelid, optic nerve, and retro-orbital space). Western studies have reported the posterior segment as the most common site of predilection whereas orbital adnexa is the most common site in Indian studies [1,9].

In the orbital adnexal form, the extra-ocular muscles are most readily involved, followed by the subconjunctival space [10]. Amongst the extra-ocular muscles, the superior rectus has been reported as the most common site [2]. In a study performed on 36 cases of ocular cysticercosis, subconjunctival mass and proptosis were reported to be the most common presenting complaints [11]. Patients with involvement of extra-ocular muscles present with non-specific symptoms like recurrent swelling, lid edema, disturbed ocular motility, and proptosis. Many such patients are misclassified as pseudotumor and are initiated on steroids which gives a temporary relief owing to its anti-inflammatory effect [3]. In our case, the patient had an unusual history of recurrent left eyelid swelling for two years with associated diplopia and difficulty in downward gaze. She was being treated conservatively for these complaints before the diagnosis.

The intra-ocular form is usually readily diagnosed owing to its obvious visibility on a basic slit-lamp examination and the commonest symptom being vision loss, the diagnosis of extra-ocular muscle cysticercosis remains tricky and requires a high clinical index of suspicion [12]. Usually, the clinical findings are non-specific and need to be supplemented by either serological or radiological investigations. Serological tests like indirect hemagglutination, indirect immunofluorescence, and immune electrophoresis may show a false positive report. Hence, imaging modalities like ultrasonography or cross-sectional imaging (CT scan/MRI Scan) are most accurate for establishing the diagnosis [13].

Past research studies have demonstrated that ultrasonography and CT Scan, both have similar diagnostic performance in cases of ocular cysticercosis [14]. However, ultrasonography is considered the initial modality of evaluation because it is relatively cheap, readily available, and possesses no risk of radiation to the patient. In cases of extraocular muscle involvement ultrasound usually reveals an enlarged muscle with a scolex within. Following the diagnosis, it is employed for monitoring the response of the treatment by repeating after every two weeks till the scolex is no longer visualized [15].

Other imaging modalities like CT and MR have high diagnostic potential but they have their own limitations in terms of cost, availability, and the need for an expert radiologist [14]. On CT Scan, the lesion is usually seen as a hypodense mass with focal hyperdensity representing the scolex. The involved muscle appears bulky and surrounding inflammatory stranding may be present. In the calcified nodular stage, an isolated calcified granuloma-like lesion may be seen with or without any inflammatory changes [10]. On MRI Scan, a well-defined round to oval cystic lesion is seen within the involved extra-ocular muscle, with a tiny enhancing mural nodule within the cyst. A study done on 161 cases of neurocysticercosis, revealed the better performance of MRI over CT for the detection of scolex within the cyst [16].

In both CT and MRI Scans, brain sections need to be evaluated thoroughly to rule out the possibility of lesions of neurocysticercosis. In our case, no evidence of concurrent neurocysticercosis was noted. In a previous study performed on 20 patients with orbital cysticercosis, only one patient had associated lesions in the brain parenchyma [2].

A study done by Puri and Grover on 21 cases of orbital cysticercosis revealed 10 cases with involvement of medial rectus, seven involving the superior rectus, and four involving the lateral rectus. Involvement of the inferior rectus or the oblique muscles wasn't reported [17]. However, another study reported eight patients with involvement of superior rectus out of a total of 35 cases [3]. In our case also the involvement of the superior rectus was noted.

Conservative management in the form of an antihelminthic agent under cover of a steroid drug is initially tried before resorting to surgical management. However, a close follow-up is recommended during the course of the treatment for the possibility of any adverse events [18]. Surgical excision of the cyst has also been described, though the method itself is challenging and also poses a risk of increased fibrotic response and consequent restricted ocular motility [4].

The Sotelo et al. study recommends oral albendazole to be prescribed at a rate of 15 mg/kg body weight which has shown significant improvement in patient condition especially in cases of extraocular cysticercosis [19]. Even though praziquantel is also effective against cysticercosis, albendazole is usually preferred due to its high elimination rate [3]. In our case as well albendazole was used for medical management.

## Conclusions

It becomes important to report this case because of the young age of the patient and the significant longevity of the complaints without any definite cure. Isolated extraocular muscle involvement in cysticercosis is fairly rare and usually presents with a wide range of non-specific complaints. In refractory/recurrent cases especially in endemic areas, a possible suspicion of orbital cysticercosis needs to be considered. An in-depth clinical history with respect to diet and travel history can aid in coming down to the diagnosis. Imaging modalities in the form of B-scan ultrasonography and cross-sectional imaging (CT scan and MRI scan) prove to be crucial in terms of confirmation of diagnosis and guiding further management.
